# The role of TEVAR in the management of a recurrent aorto-gastric fistula

**DOI:** 10.1093/jscr/rjy014

**Published:** 2018-02-15

**Authors:** Terri-Ann T Russell, Pranitha Pinnamaraju, Maher Hamish

**Affiliations:** Vascular Surgery Department, Northampton General Hospital, Northampton NN1 5BD, UK

## Abstract

An aorto-gastric fistula is a catastrophic and rare cause of an upper gastrointestinal bleed. The diagnosis requires a high index of suspicion and expedient management as any delay in each of these component, will be to the detriment of the patient. We report a case of a patient with two episodes of this rare event, with haemodynamic compromise, 15 years after having had a trans-hiatal oesophagectomy for an adenocarcinoma of the oesophagus who presented on both occasions. He had thoracic endo-vascular aortic repair (TEVAR) on both presentations and survived. This case exemplifies the fact that while TEVAR is a good bridging therapy for the management of an aorto-enteric fistula. It however should not be considered as the definitive management for patients who are operable or patients who do not have prohibitive surgical risk.

## INTRODUCTION

Despite the first case of an aorto-enteric fistula (AEF) being discovered in 1818; its rare incidence results in a low index of clinical suspicion. Its usual presentation is that of sudden onset of massive haematemesis; and this along with it being rare often results in a late or missed diagnosis and hence a high mortality.

The definitive role of thoracic endo-vascular aortic repair (TEVAR) in the management of AEFs is still undefined. It however provides a rapid means to achieve haemodynamic stability and is associated with low morbidity and mortality. Despite the advantages of TEVAR the current standard of care is still open surgery which involves replacement of the diseased segment of aorta and oesophagus and excision of the fistula. The mortality of open repair is usually high and hence a technique which is definitive with a low morbidity and mortality is still being sought after.

## CASE REPORT

**Figure 1:**
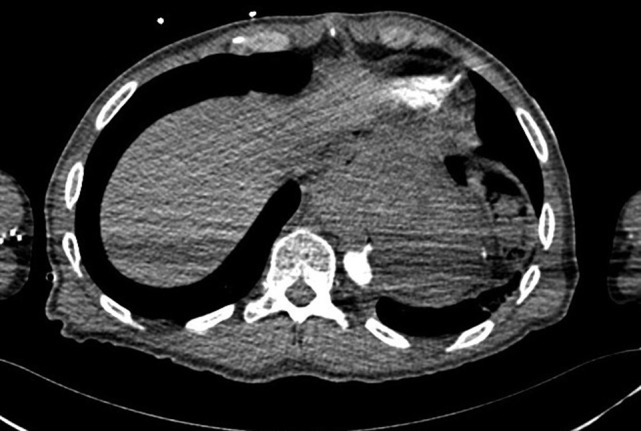
CT aortogram showing the extravasation of contrast into the stomach.

The purpose of this case report is to describe the clinical course of a 57-year-old male who presented with acute onset of massive haematemesis and hypovolaemic shock. His medical history was remarkable for a trans-hiatal oesophagectomy and gastric pull-up for adenocarcinoma 15 years prior. After initial fluid resuscitated, oesophago-gastro-duodenoscopy (OGD) was performed which was complicated by cardiac arrest. He was intubated and fluid resuscitated until haemodynamically stable. Computed tomography aortography (CTA) demonstrated an aorto-oesophageal fistula (Fig. 1). A rapid decision was made to proceed with a TEVAR limited to that segment of aorta using Cook Zenith Alpha 24/105 stent graft. The procedure was successful. The patient was later offered definitive procedure, which he refused. Follow-up OGD after 4 weeks revealed a gastric ulcer, with no abnormal cells on histology, for which he was placed on high dose proton-pump inhibitor.

**Figure 2:**
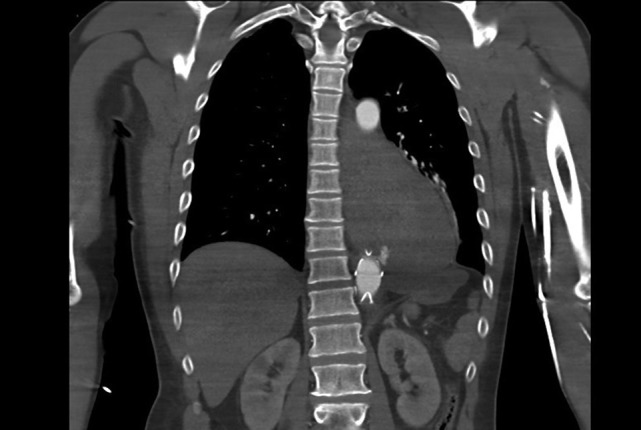
CT aortogram showing the extravasation of contrast from the aorta just proximal to the stent graft.

He re-presented with hypovolaemic shock and massive haematemesis four months later. A diagnosis of AEF was again confirmed on CTA (Fig. 2), just proximal to the previous aortic stent graft. He again had emergency percutaneous TEVAR covering the descending aorta from the level just below the left subclavian artery to just proximal to the celiac artery. Again, he was offered definitive surgery, but he was still not keen to have this at that time.

**Figure 3:**
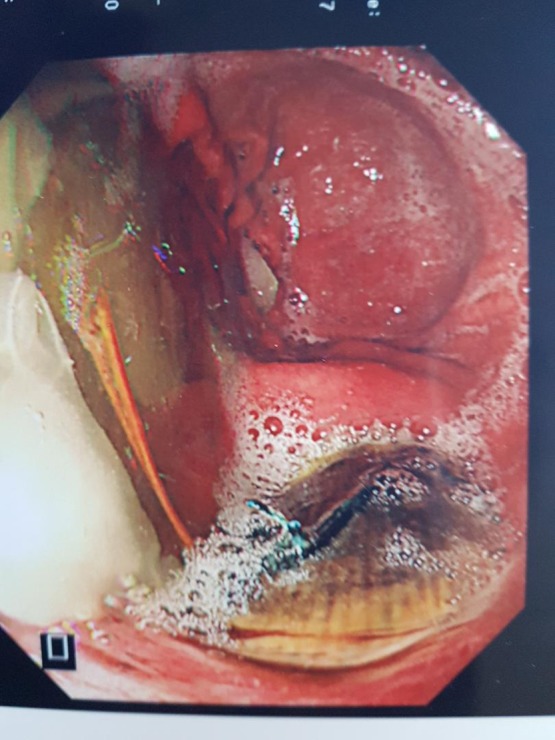
Endoscopic view of aortic stent graft in base of gastric ulcer.

Two months later, repeat OGD revealed a persistent gastric ulcer with a visible segment of aortic stent graft in its base (Fig. 3). He, then accepted the option of definitive repair. He was immediately transferred to a cardio-thoracic tertiary centre where he underwent a thoracotomy, repair of aorto-gastric fistula with primary stomach repair and thoracic and abdominal aorta replacement with a Dacron graft using left heart bypass. He made a good recovery and had no complications. His aortic stent-graft culture grew Candida albicans and vancomycin-sensitive and vancomycin-resistant enterococcus.

## DISCUSSION

Even though the first case of a successful repair of an AEF was performed by Jones [1], the mortality remains high and some series quote as high a mortality as 81% [2]. The principle behind the management is rapid control of bleeding, reconstruction of the aorta and oesophagus and prevention of mediastinal contamination. This operation is predominantly done through a thoracotomy with the use of cardio-pulmonary bypass.

Rapid diagnosis is essential as there is typically a narrow window before exsanguination occurs. Conservative management of watchful waiting is not an option as this is associated with 100% fatality [3]. Clinically these patients present with massive haematemesis, however, Chiari described a triad of chest pain, fever and haematemesis, of which only few patients present with this constellation of symptoms [4].

Majority (54%) of AEF are due to an aortic aneurysm; foreign body and advanced cancer respectively are the second and third most common causes. In this case the pathology was a gastric ulcer. There are case reports of patients who had AEF early after Sweet’s oesophagectomy [5] but there is no mention in the literature of such a late presentation, 15 years later post oesophagectomy.

The use of endo-vascular strategies to treat a fistula was first report by Chuter *et al.* [6], who used a stent graft to exclude an aorto-bronchial fistula . Even though there are no guidelines to address the use of TEVAR in the management of AEFs its use in other aortic pathologies such as aortic dissections and aneurysms, and the benefits of low morbidity and mortality associated with these procedures favour its use when available to rapidly salvage a patient exsanguinating due to an aorto-oesophageal fistula [5]. The benefits of TEVAR as a first-choice procedure in these cases are; the avoidance of a thoracotomy, aortic cross-clamp and cardio-pulmonary bypass and their associated complications, especially in a haemodynamically compromised patient.

TEVAR however has its known complications such as: embolic events example stroke; paraplegia and visceral ischaemia. Its overall safety profile is nevertheless quite impressive with mortality of 1.9–2.1% compared with 11% mortality with open surgery for descending thoracic aorta aneurysms [7, 8]. Due to its excellent results TEVAR is safe, particularly in patients who have high surgical risks with multiple comorbidities. It is therefore by extrapolation a good procedure for extremist patients with AEF.

There is however on-going debate about the use TEVAR in a contaminated field, and of the risk of mediastinitis and the morbidity and mortality associated with an infected stent graft post aortic stenting for AEF. For these reasons, many case reports suggest the use of TEVAR being only temporary, as a first-choice life-saving measure, and that a definitive elective surgical procedure is to be done once the patient recovers from the ordeal of the massive bleed. This extrapolation however is just from case reports as there are no randomized study to prove this. There is also a query of whether these patients should receive lifelong broad-spectrum antibiotics [9], there is however no consensus on the duration of antibiotic therapy post TEVAR.

The role of TEVAR in the management of aorto-enteric fistulae is inconclusive. It is nonetheless an excellent bridging therapy and a possible definitive therapy for patients with haematemesis who are high risk for surgery or have advanced cancer respectively.
